# Socioeconomic inequities impacting complete continuum of maternal healthcare service utilisation over time in Ethiopia

**DOI:** 10.1007/s10754-025-09401-x

**Published:** 2025-08-23

**Authors:** Ayal Debie, Molla M. Wassie, Annabelle Wilson, Claire T. Roberts, Jacqueline H. Stephens

**Affiliations:** 1 College of Medicine and Public Health, Flinders Health and Medical Research Institute, Flinders University, Adelaide, Australia; 2 Department of Health Systems and Policy, Institute of Public Health, University of Gondar, Gondar, Ethiopia

**Keywords:** Equity, Continuum, Maternal healthcare service, Ethiopia

## Abstract

**Objective:**

To quantify socioeconomic inequities of complete continuity of maternal healthcare service over time using Ethiopian Demography and Health Survey data from 2011 to 2019.

**Methods:**

A total of 10,768 women who had at least one antenatal care visit during their most recent childbirth were included for the analysis. Concentration index and concentration curve were used to assess wealth-based inequities. A generalized linear model with binomial distribution and a logit link function was used to decompose the Erreygers concentration index and measure each determinant’s contribution.

**Results:**

Complete continuum of maternal health service utilization in 2011, 2016, and 2019 among the wealthiest women were 25.9%, 33.7%, and 50.8%, respectively. In contrast, the corresponding continuum of maternal health service utilisation was 3.0%, 6.1%, and 11.2% among the lowest wealth categories. The Erreygers concentration indices of complete continuum of maternal health service utilization in 2011, 2016, and 2019 surveys were 0.203, 0.195, and 0.311, respectively, with the highest inequity observed in 2019. Concentration curves in each survey showed a pro-rich distribution of complete continuum of maternal health service utilisation. A unit percentage change in women’s education, early initiation of antenatal care, being informed about pregnancy-related complications, and blood pressure monitoring during pregnancy increased their probability of completing continuum of maternal health service utilization. Specifically, a 1% increase in the proportion of secondary or higher education corresponded to a 0.02%, 0.01%, and 0.07% increase in the probability of completing continuum of maternal health service utilisation in 2011, 2016, and 2019, respectively. Conversely, in 2011, a 1% increase in the proportion of rural women and those with more than four parities led to a 0.11% and 0.05% decrease in the probability of completing continuum of maternal health service utilisation, respectively.

**Conclusion:**

Complete continuum of maternal health service utilization was more likely amongst women without disadvantage, demonstrating wealth-based inequities in continuum of maternal health service utilization continue in Ethiopia. In this analysis, continuum of maternal health service utilisation remains inelastic across all surveys highlightsits the service is an essential form of care for women. Provision of maternal healthcare services targeting women from low household wealth status, residing in rural communities, and uneducated women must be prioritised by policymakers.

## Significance

What is already known on this subject?

What this study adds?

Place of residence and household wealth status play significant roles in determining the continuity of maternity care. Previous evidence on socioeconomic inequity of continuity of maternity care over time was limited in Ethiopia.

This study showed that the progress toward universal maternity care remains insufficient to achieve the 2030 target, even among the wealthiest rural women who have had at least one antenatal care visit. Continuity of maternity care had pro-rich distribution throughout the surveys from 2011 to 2019, with the highest inequity observed in 2019. In addition, this study offers methodological insights and encouraging future researchers to assess wealth-based inequities over time for binary health outcome using line graph, concentration curve (CC), and Erreygers concentration index (ECI). The study also gives additional methodological insights on how decomposition of concentration index has been conducted to assess wealth-based service uptake inequities (in this case, complete continuum of maternal health service utilization). Importantly, we used ECI to compare the inequities across surveys which is the preferred method of measuring inequities in binary health outcome.

## Introduction

In 2020, the global Maternal Mortality Ratio (MMR) was 223 maternal deaths per 100,000 live births and 545 deaths per 100,000 live births in sub-Saharan Africa (SSA) (World Health Organization, 2023). Between 2000 and 2020, the average annual reduction rate (ARR) in the global MMR was 2.1%, however, to achieve the Sustainable Development Goal (SDG) about 11.6% average ARR is required for the remaining ten years (2021 to 2030) (World Health Organization, 2023). Realising MMR of less than 70 maternal deaths per 100,000 live births by 2030 is the global target set by the United Nations (World Health Organization, 2015). However, Ethiopia still has high maternal mortality, with approximately 267 maternal deaths per 100,000 live births in 2020 (World Health Organization, 2023). Improving access, coverage, and quality of integrated service-delivery packages throughout the continuum of care saves the lives of women and children (Kerber et al., 2007).

Continuum of maternal healthcare services (CMHS) is defined as the uninterrupted access and utilization of healthcare services a woman receives throughout her pregnancy, childbirth, and postpartum period, including antenatal care (ANC), delivery assisted by a skilled birth attendant, and postnatal care (PNC). This CMHS utilisation varies across geographic areas and socioeconomic groups (Asratie et al., 2020; Dennis et al., 2019). Just a quarter (25%) of women in South Asia and 14% in SSA receive complete CMHS (Singh et al., 2016; Zelka & Yalew, 2019). Studies conducted in Ghana and Tanzania show only 8% and 10% of women received complete CMHS, respectively (Mohan et al., 2017; Yeji et al., 2015). In Kenya, one-third (34%) of women in their recent pregnancy received a complete CMHS (Mwangi et al., 2018). A meta-analysis in Ethiopia showed only 25.5% of women had completed CMHS in 2022 (Addisu et al., 2022).

Equity in health is the absence of unfair and avoidable differences in health among different population groups to attain their highest level of health, regardless of social, economic, or demographic factors (World Health Organization, 2012). Ethiopia is a nation with low coverage of access to essential health services and health service utilisation with high sub-national disparities (FMOH - Ethiopia, 2017). Building an equitable and acceptable standard of health service system to reach all segments of the population is among the pillars of the health policy of Ethiopia, (FMOH - Ethiopia, 1993) to achieve Universal Health Coverage (UHC). In Ethiopia, the second Health Sector Transformation Plan (HSTP II) primarily emphasises in improving equity and quality of healthcare services (Ministry of Health Ethiopia, 2021).

Over the past two decades, various community-based healthcare interventions have been also employed in Ethiopia. For example, the Health Extension Program (HEP) in 2003 and the Health Development Army (HDA) in 2011 were introduced to provide equitable community-based essential healthcare services, with a particular focus on maternal and child healthcare services (FMOH - Ethiopia, 2006; Rieger et al., 2019). The Health Extension Program in Ethiopia is among the strategies aiming to achieve UHC through a primary health care (PHC) approach (Wang et al., 2016). In addition, the user fee-free maternal healthcare service policy was another intervention to address equitable access and ensure that financial constraints do not hinder pregnant women from seeking essential maternal healthcare services (Marye et al., 2023). However, socioeconomic and obstetric-related factors still influence the use of maternal healthcare services (Mekonnen & Mekonnen, 2003). In healthcare, equity needs to ensure universal access to care for everyone. However, there is only limited evidence available on the equity of complete CMHS utilisation in Ethiopia. Therefore, this study aimed to assess the socioeconomic inequities and contributors to complete CMHS utilisation over time using EDHS 2011 to 2019 data using a decomposition analysis.

## Methods and materials

### Study design and settings

We used cross-sectional secondary data to assess socioeconomic inequities of the complete CMHS utilization in Ethiopia using the Ethiopian Demographic and Health Survey (EDHS) from 2011 to 2019. We used the former regions of Ethiopia, including Tigray, Afar, Amhara, Benishangul-Gumuz, Gambela, Harari, Oromia, Somali, and South Nation, Nationality, and People (SNNP), and two city administrations (Addis Ababa and Dire-Dawa) for this study.

### Sample and sampling procedures

**Fig. 1:**
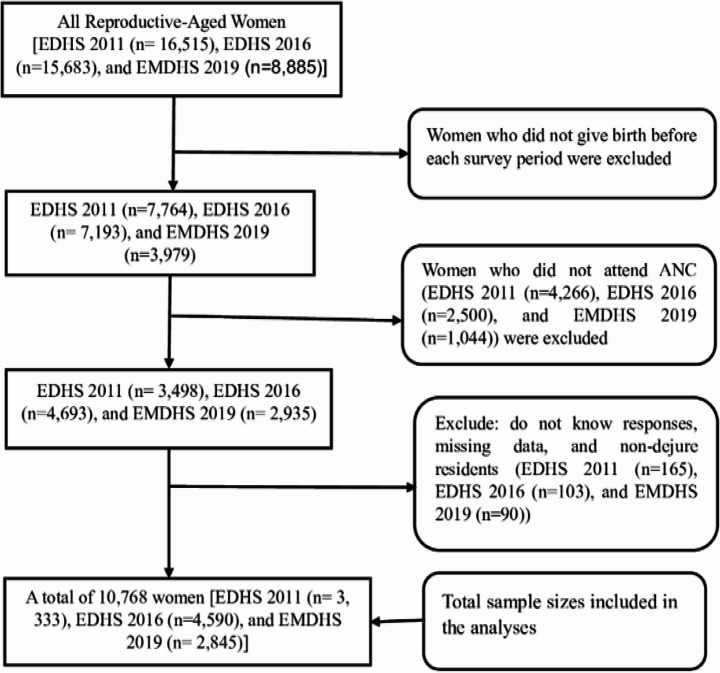
Final sample sizes included for the analyses from the Ethiopian Demography and Health Survey (EDHS) 2011 to Ethiopian Mini-DHS 2019

In this study, all women who had at least one ANC visit for their recent childbirth during the survey period from EDHS 2011 to 2019 were included in the study population. We used publicly available data for this study (available from: https://www.dhsprogram.com/data/dataset_admin/login _main.cfm). In each EDHS from 2011 to 2019, each region of Ethiopia was stratified into urban and rural areas, resulting in a total of 21 sampling strata. Samples of enumeration areas (EAs) were independently selected within each stratum using a two stage sampling procedure. In the first stage, a total of 624 EAs (437 rural and 187 urban) in 2011, 645 EAs (443 rural areas and 202 urban) in 2016, and 305 EAs (93 urban and 212 rural) in 2019 were selected with proportional to EA size. In the second stage, household listing operations were performed in all selected EAs. On average, 27 to 32 households per cluster were selected proportional to the cluster size using systematic sampling. Relevant variables were extracted from the Individual Record (IR) file of EDHS 2011 to 2019 datasets. The data cleaning process involved a number of steps to identify eligible women within each dataset. First, we excluded women who had not given birth before the survey period. Second, we removed those who had not attended at least one ANC visit. Third, we excluded records with missing data, including “don’t know” responses and women who were not *de jure* residents. Finally, a total weighted samples of 10,768 (3,333 in 2011, 4,590 in 2016, and 2,845 in 2019) were included in the study (Fig. 1). The details of the sampling procedures described in the EDHS reports (CSA, 2011, 2016, 2019).

### Study variables and operational definitions

Complete CMHS was the outcome variable. CMHS is an integrated and seamless provision of care and services to women throughout their pregnancy, childbirth, and postpartum. Thus, completion of the CMHS was defined as attending a minimum of four ANC visits, giving child birth at a health facility, and receiving at least one postnatal care (Wang & Hong, 2013). We categorized the responses for each of the three maternal healthcare services as either “yes” or “no” and coded them as “1” and “0”, respectively. If a woman reported “no” for one or more of these three maternal healthcare services, we considered that she could not use complete CMHS. In contrast, a woman who reported “yes” to all three maternal healthcare service components was supposed to have completed CMHS (Wang & Hong, 2013). A woman was also considered to have a postnatal care visit if a woman had a health check up before discharge or after discharge following health facility birthing/after delivery at home.

Socio-economic characteristics were included as covariates, such as women’s age, religion, maternal education, place of residence (urban vs. rural), current marital status, geographic region, age at first birth, and household wealth index. Additional covariates related to obstetric history included the timing of the first ANC visit, whether urine and blood samples were taken during pregnancy, blood pressure monitoring during pregnancy, and caesarean delivery for the most recent childbirth. Ownership of a radio or television and access to electricity were also considered.

### Data management and analyses

Stata software was used to analyse the data (Stata Corp. 2023. Stata Statistical Software: Release 18. College Station, TX: Stata Corp LLC). Before any statistical analyses, the data were weighted using survey weight to adjust differences in the probability of selection and maintain the survey’s representativeness. Concentration curve (CC) and concentration index (CI) are the main measures used to assess health inequality (Wagstaff et al., 1991). Concentration curve is an X-Y plot graphical representation that helps to visualise the distribution of a particular variable across different levels of wealth groups (O’Donnell et al., 2007; Wagstaff et al., 1991). The x-axis represents the cumulative percentage of women, ranked by their wealth index from the poorest to the richest, and the y-axis represents the cumulative percentage of complete CMHS utilization. The line of equality represents a situation where CMHS utilization is equally distributed across wealth groups. Any deviation from this line indicates the presence of inequality. The CC was drawn using the *glcurve Stata* command (O’Donnell et al., 2007).

Concentration index is twice the area between the concentration curve and the line of equality (the 45-degree line). Concentration index summarises the degree to which a health outcome (CMHS utilization) is concentrated across wealth groups. The value of CI ranges between − 1 and 1. The values − 1 and 1 denote absolute inequality, and a zero concentration index value represents equitable service distribution. A positive value shows that the health outcome (CMHS utilization) is concentrated among wealthier groups, and a negative value indicates the concentration is among low-wealth groups. In measuring health inequalities, the magnitude of the concentration index for a health outcome conveys the relative degree of concentration of the health outcome (proportion of complete CMHS utilization) among poor or rich groups.

Concentration index is estimated from the covariance between health outcome and the socioeconomic rank of the participants or households, multiplied by two, and then divide the whole expression by the mean of the health outcome variable (Wagstaff et al., 1991).

$$\:CI=2*\frac{Cov\left(yi,ri\right)}{\mu\:}$$

CI is the concentration index for complete CMHS utilization, µ is the mean of the health outcome (in this case, proportion of complete CMHS utilization), y_i_ is the health outcome of the i^th^ participant, cov is covariance, and r_i_ is the fractional rank of the i^th^ household (woman) in the wealth distribution.

The range of values for the standard concentration index depends on the mean (µ) of the outcome variable (proportion of CMHS in this case). The lower and upper bounds can be (𝜇 − 1) and (1 − 𝜇) instead of − 1 and + 1, respectively (Wagstaff, 2005). For binary outcome, Erreygers concentration index (ECI) is preferable over the standard concentration index. The ECI is a modification of the standard concentration index when the outcome variable has binary alternatives (Erreygers, 2009).

$$\:\text{E}\text{C}\text{I}=4\text{*}{\upmu\:}\text{*}\text{C}\text{I}\:$$

ECI is Erreygers concentration index, µ is the mean of the health outcome (proportion of complete CMHS utilization), CI is generalized concentration index. We used *Conindex* Stata command to measure concentration index (O’Donnell et al., 2016).

### Decomposition of concentration index

We used a Generalized Linear Model (GLM) with binomial distribution and logit link function as a nonlinear regression model to decompose the ECI and to determine the contribution of each determinant (Yiengprugsawan et al., 2010). In this case, a linear additive regression model for health outcome variable y, with a set of k determinants (X_k_) is shown as follow:

$$y=\alpha+\sum_k\beta kXk+\epsilon$$

βk denotes the coefficients and ϵ is an error term. To determine the contribution of each determinant and the concentration index for y (CIy) of the above equation can be rewritten as:

$$CIy=\sum_k\left(\frac{\beta k\overline Xk}\mu\right) C\kappa+\frac{GC\in}\mu$$

The coefficient (β_k_) is marginal or partial effects of determinant X_k_, X̅_k_ is the mean of the determinant X_k_, µ is the mean of the health outcome (proportion of complete CMHS utilization), C_k_ is the concentration index of the determinant X_k_, GCϵ is the generalized concentration index for the error term (residual component).

The overall wealth-based inequities depend on the elasticity and the extent of unequal distribution of each determinant across socioeconomic groups (concentration index). Elasticity indicates the impact of each determinant on the probability of the desired health outcome (complete CMHS utilisation), that is, how much change in the proportion of complete CMHS utilization is associated with a unit percentage change in the explanatory variable. The positive sign of the elasticity measure indicates an increase in the explanatory variable; the demand for complete CMHS utilisation also increases. The magnitude of the elasticity shows that the demand response is either small or large (Ringel et al., 2002).

## Results

### Study characteristics

This study included 10,768 (3,333 in 2011, 4,590 in 2016, and 2,845 in 2019) women who had at least one ANC visit in their recent childbirth, with an overall response rate of 96.8%. Of these, 20.9% of women aged above 34 years participated in 2019. Over the study period, the proportion of women who did not attend any education and attended secondary or higher education in 2016 were 54.4% and 12.6%, respectively. Approximately 75.3%, 65.8%, and 72.5% of women in 2016 had blood pressure assessments and gave urine and blood samples during pregnancy, respectively. Only 26%, 32.4%, and 37.5% of pregnant women had started ANC at their first trimester in 2011, 2016, and 2019, respectively. The proportion of the richest was 26.4% in 2019, while the proportion of women with the poorest household wealth status was 13.9% (Table 1).

**Table 1:** Study participants’ characteristics among women who had at least one ANC visit for their recent delivery from 2011 to 2019 in Ethiopia (*n* = 10,768)

Characteristics	Categories	EDHS 2011 (*n* = 3,333)	EDHS 2016 (*n* = 4,590)	EMDHS 2019 (*n* = 2,845)
Age of women in years	< 20	4.21	5.24	5.36
	20–34	72.05	72.88	73.72
	≥ 35	23.74	21.88	20.92
Age at first childbirth in years	< 15	6.19	5.66	14.22
	15–19	55.04	56.10	47.83
	≥ 20	38.77	38.25	37.96
Sex of household head	Male	82.67	85.64	87.25
	Female	17.33	14.36	12.75
Place of residence	Urban	26.55	18.20	29.77
	Rural	73.45	81.80	70.23
Religion	Orthodox	46.56	42.17	41.44
	Catholic	1.08	0.91	0.22
	Protestant	21.68	22.20	27.24
	Muslim	29.14	33.09	30.21
	Others	1.54	1.63	0.89
Maternal education	No education	53.03	54.36	43.89
	Primary education	37.16	33.08	39.73
	Secondary/Higher	9.81	12.57	16.38
Antenatal care visit in 1 st trimester	No	74.04	67.58	62.51
	Yes	25.96	32.42	37.49
Parity	1	21.11	22.77	23.00
	2–4	44.90	44.26	47.33
	≥ 5	33.99	32.97	29.67
Current marital status	Unmarried	8.69	6.12	5.60
	Married	91.37	93.88	94.40
Family size	< 5	33.28	35.63	37.26
	≥ 5	66.72	64.37	62.74
Being informed pregnancy-related complications	No	79.51	55.28	39.56
	Yes	20.49	44.72	60.44
Caesarean delivery	No	95.94	96.46	92.04
	Yes	4.06	3.54	7.96
Blood pressure measured during pregnancy	No	28.27	24.71	11.77
	Yes	71.73	75.29	88.23
Took urine samples during pregnancy	No	59.28	34.22	25.96
	Yes	40.72	65.78	74.04
Took blood samples during pregnancy	No	46.05	27.53	20.89
	Yes	53.95	72.47	79.11
Wealth index	Poorest	12.77	16.74	13.87
	Poorer	17.57	19.80	19.82
	Medium	18.56	20.91	20.24
	Richer	20.66	20.12	19.64
	Richest	30.44	22.42	26.43
Owned television	No	85.30	85.55	78.37
	Yes	14.70	14.45	21.63
Owned radio	No	50.31	69.23	69.35
	Yes	49.69	30.77	30.65
Access to electricity	No	72.80	76.20	63.37
	Yes	27.20	23.80	36.63
Region	Tigray	10.02	10.15	9.32
	Afar	0.80	0.77	1.11
	Amhara	22.78	22.56	23.91
	Oromia	36.66	34.04	37.05
	Somali	1.46	2.51	2.19
	Benishangul-Gumuz	1.12	1.20	1.33
	SNNP	20.28	23.58	19.44
	Gambela	0.53	0.32	0.57
	Harari	0.34	0.29	0.31
	Addis Ababa	5.53	3.98	4.19
	Dire Dawa	0.47	0.61	0.60

*Footnote: EDHS: Ethiopian Demography and Health Survey*

### Continuity of maternity care across wealth categories

**Fig. 2:**
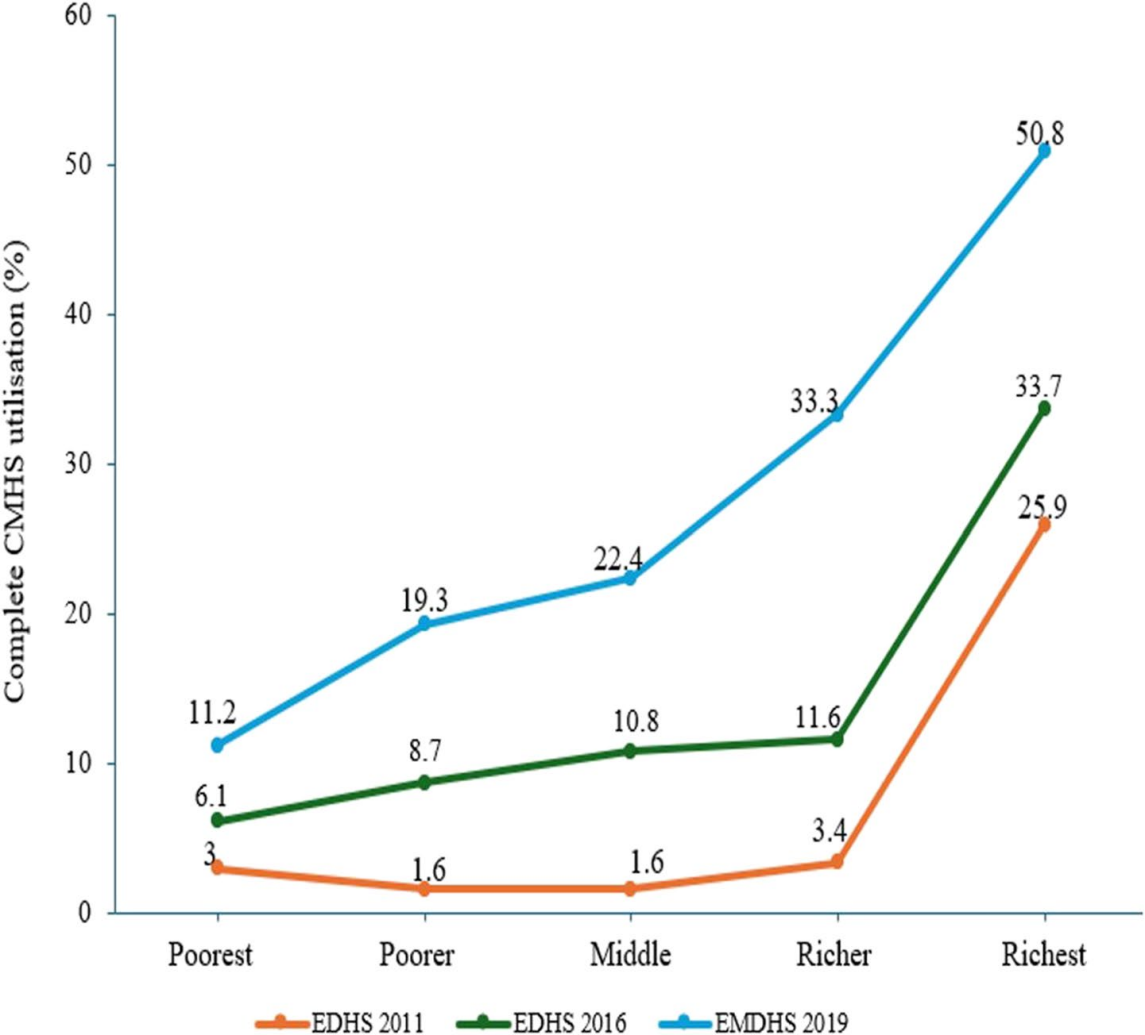
Complete CMHS utilization across wealth categories among women who had at least one ANC visit for their most recent childbirth overtime in Ethiopia

This study showed that each broad component of the CMHS increased from 2011 to 2019. Specifically, complete CMHS utilization among women with at least one prenatal care visit for their most recent birth was 9.6% (95%CI: 8.4, 10.9) in 2011 and 29.9% (95%CI: 27.3, 32.6) in 2019. The prevalence of complete CMHS utilisation among the wealthier and wealthiest groups was remarkably higher than the middle, low, and lowest household wealth categories in every survey period. For example, about 33.3% and 50.8% of women from the wealthier and wealthiest households in 2019 used CMHS compared with lowest, lower, and middle wealth group women with a corresponding proportion of 11.2%, 19.3%, and 22.4%, respectively. Besides, the lowest proportion of complete CMHS utilization was reported among women from the poorer (lower) and middle-wealth groups in 2011. More specifically, the highest complete CMHS utilization was observed among women from richer (wealthier) and richest (wealthiest) households throughout the surveys (Fig. 2). The prevalence of each component of CMHS utilisation was also highest among the wealthier and wealthiest groups than the middle, low, and lowest household wealth categories in every survey period (Table 2).

**Table 2:** Maternity care among women who had at least one ANC visit for their recent birthing across wealth categories from 2011 to 2019 in Ethiopia

Survey period	Maternity care components	Use (%)	Use (%)	Use (%)	Use (%)	Use (%)	Overall utilisation (%)
		Poorest	Poorer	Middle	Richer	Richest	
2011	ANC4+	32.6	34.8	36.5	45.7	60.7	45.0
	HF delivery	5.6	5.6	5.9	12.5	59.5	23.5
	PNC	7.7	7.2	4.7	11.2	39.5	17.5
	CMHS	3.0	1.6	1.6	3.4	25.9	9.6
2016	ANC4+	39.0	44.8	45.4	54.3	68.3	51.1
	HF delivery	24.4	34.5	35.9	42.5	81.5	45.3
	PNC	15.5	18.7	21.2	22.8	47.4	26.0
	CMHS	6.1	8.7	10.8	11.6	33.7	14.9
2019	ANC4+	41.5	54.2	50.4	58.9	75.1	58.1
	HF delivery	38.6	52.3	55.7	71.4	88.4	64.4
	PNC	29.5	33.5	41.6	52.5	63.6	46.3
	CMHS	11.2	19.3	22.4	33.3	50.8	29.9

***Footnote***: *ANC: Antenatal care; CoC: continuum of care; HF: Health facility; PNC: Postnatal care*

**Fig. 3:**
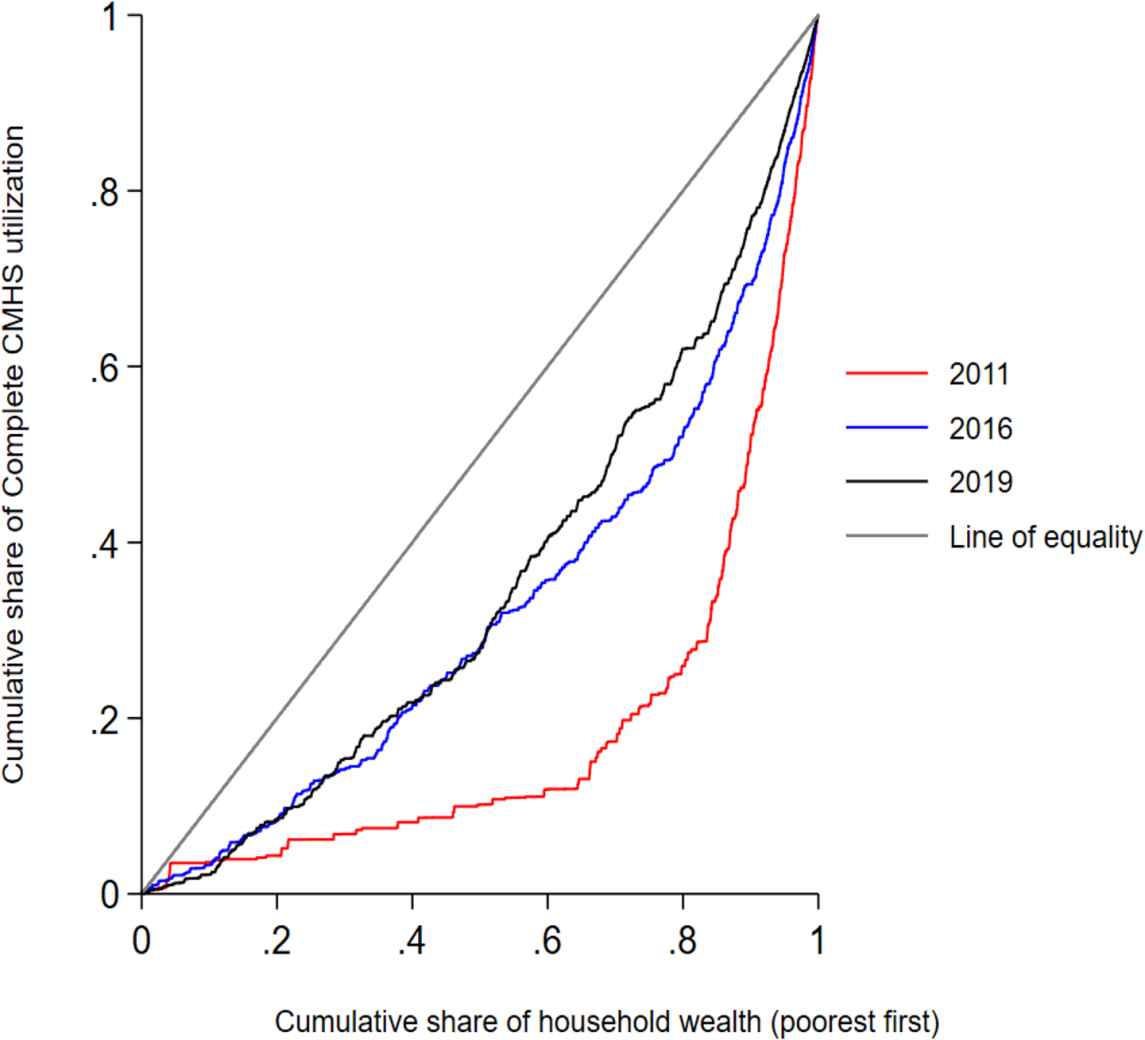
CCs of complete CMHS utilization among women who had at least one ANC visit for their most recent childbirth from 2011 to 2019 in Ethiopia

The Erreygers concentration index was above zero for each survey and showed significant association with wealth status throughout the surveys. The ECI of complete CMHS utilization across EDHS 2011, 2016, and 2019 surveys were 0.203, 0.195, and 0.311, respectively, with highest inequity observed in 2019. The ECI also showed that women had a pro-rich distribution of the CMHS across household wealth categories (Table 3).The CC for complete CMHS across each EDHS survey period was below the equality line in which the proportion of complete CMHS utilization more concentrated among wealthier and wealthiest households throughout the three surveys (Fig. 3). As shown in Fig. 3, the CC further explained that the highest inequity in CMHS utilisation was observed among wealthier groups in 2011 and among lowest wealth groups in 2019.

**Table 3:** ECIs and GCIs of complete CMHS utilisation among women who had at least one ANC visit for their most recent birthing from 2011 to 2019 in Ethiopia

Survey period	Erreygers concentration index	SE	Generalized concentration index	SE
EDHS 2011	0.203*	0.023	0.531*	0.060
EDHS 2016	0.195*	0.018	0.327*	0.029
EMDHS 2019	0.311*	0.036	0.260*	0.030

***Footnote***: *:P-value < 0.05; SE: Standard error

### Continuity of maternity care across place of residence

**Fig. 4:**
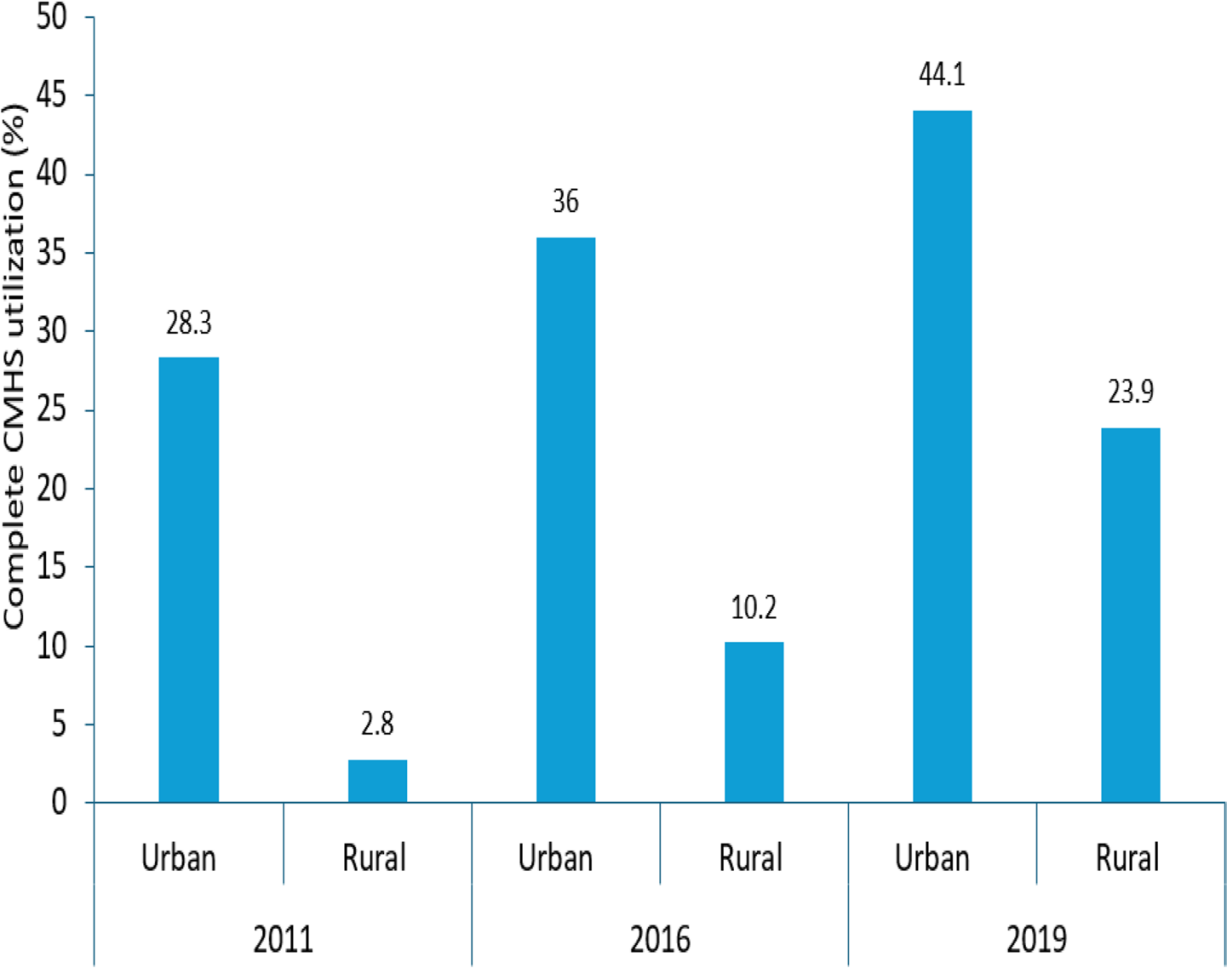
Complete CMHS utilisation by women’s place of residence among women who had at least one ANC visit for their most recent childbirth from 2011 to 2019 in Ethiopia

This study showed that urban women had consistently higher utilisation of continnum of maternity care compared to the rural women across each survey from 2011 to 2019. Specifically, we observed that 28.3%, 36.0%, and 44.1% of urban women received complete CMHS in 2011, 2016, and 2019, respectively. In contrast, only 2.8%, 10.2%, and 23.9% of rural women received CMHS in 2011, 2016, and 2019, correspondingly (Fig. 4).

### Decomposition analyses

In this study, women attending secondary or higher school, having ANC in the first trimester, being informed about pregnancy-related complications, measuring blood pressure during pregnancy, and having caesarean delivery were positively associated with complete CMHS utilization in each survey. In addition, taking blood samples for maternal healthcare assessment during pregnancy in 2011 and 2016, having their own television in 2011 and 2019, and household wealth status in 2016 and 2019 were also positively associated with complete CMHS utilization. On the contrary, rural women and women with more than four parity in 2011 and 20-34-years-old women in 2016 were negatively associated with complete CMHS utilization. However, women over 20 years-old in the 2019 survey were positively associated with CMHS utilization (Table 4). In this decomposition analysis, we observed that the relationship between the binary health outcome (CMHS) and the independent variables vary across the surveys. Women’s place of residence and access to ANC check-ups such as blood pressure and blood sample assessments in 2011, household wealth status, being informed about pregnancy complications, early ANC visits, and ownership of television in the survey periods of 2016 and 2019 were the leading contributors. Specifically, women’s place of residence in 2011, measuring blood pressure in 2016, and having their own TV in 2019 had the highest contribution to CMHS utilisation inequities with corresponding percent contribution of 36.0%, 33.0%, and 31.2%, respectively. In this analysis, we also assured that CMHS remained inelastic across all survey periods, with elasticity values consistently falling between − 1 and 1. Additionally, a unit percentage increase in factors such as attainment of primary or higher education, early in ANC in the first trimester, being informed about pregnancy-related complications, caesarean delivery, ownership of television, access to electricity, and taking urine and blood samples during pregnancy increased the probability of completing CMHS utilization across all surveys. For instance, a 1% increase in the proportion of secondary or higher education corresponded to a 0.02%, 0.01%, and 0.07% increase in the probability of completing CMHS utilisation in 2011, 2016, and 2019, respectively. Conversely, in 2011, a 1% increase in the proportion of rural women and those with more than four parities led to a 0.11% and 0.05% decrease the probability of completing CMHS utilisation, respectively. We also found that the proportion of the residual component contribution was negative throughout the surveys. This showed that the unobserved variables (factors that are not included in the model) counteract the contributions of wealth-based CMHS inequality driven by the observed factors (Table 5).

**Table 4:** Factors associated with the probability of complete CMHS utilization among women who had at least one ANC visit for their recent childbirth from 2011 to 2019 in Ethiopia

Variables	Category	2011	2016	2019
		Coeff (95CI%)	Coeff (95%CI)	Coeff (95%CI)
Maternal age in years	< 20 (ref)			
	20–34	0.47(−0.23, 1.18)	−0.52(−0.94, −0.10)*	0.54(0.07, 1.01)*
	≥ 35	0.59(−0.23, 1.41)	−0.40 (−0.89, 0.10)	0.83(0.29, 1.38)*
Age of women at first childbirth	< 15 (ref)			
	15–20	−0.37(−0.95, 0.21)	0.25 (−0.16, 0.67)	0.01 (−0.28, 0.30)
	≥ 20	−0.30(−0.90, 0.30)	0.48 (0.04, 0.91)*	−0.01(−0.32, 0.31)
Sex of household head	Male (ref)			
	Female	0.11(−0.19, 0.41)	0.14 (−0.07, 0.35)	0.19(−0.06, 0.43)
Residence	Urban (ref)			
	Rural	−0.95(−1.39, −0.51)*	−0.15(−0.45, 0.15)	−0.07(−0.35, 0.22)
Maternal education	No education (ref)			
	Primary	0.40(0.08, 0.71)*	0.17(−0.04, 0.38)	0.27(0.05, 0.49)*
	Secondary or higher	0.95(0.58, 1.31)*	0.51 (0.25, 0.77)*	0.55(0.27, 0.82)*
ANC visit during 1 st trimester	No (ref)			
	Yes	0.63(0.38, 0.88)*	0.53 (0.36, 0.70)*	0.61 (0.43, 0.80)*
Parity	1 (ref)			
	2–4	−0.25(−0.55, 0.06)	0.25 (0.02, 0.47)*	−0.23(−0.48, 0.03)
	≥ 5	−0.69(−1.22, −0.17)*	0.18 (−0.17, 0.53)	−0.12-0.50, 0.25)
Current marital status	Unmarried (ref)			
	Married	−0.26(−0.67, 0.15)	−0.23(−0.53, 0.08)	0.01(−0.35, 0.38)
Family size	< 5 (ref)			
	≥ 5	0.20(−0.07, 0.48)	−0.05(−0.24, 0.15)	−0.12(−0.33, 0.10)
Being informed pregnancy complications	No (ref)			
	Yes	0.64(0.39, 0.88)*	0.64(0.47, 0.81)*	0.91(0.72, 1.10)*
Caesarean delivery	No (ref)			
	Yes	1.44(1.08, 1.80)*	1.33(1.02, 1.65)*	0.74(0.44, 1.03)*
Measured BP during pregnancy	No (ref)			
	Yes	0.99(0.41, 1.58)*	1.19(0.80, 1.59)*	0.67(0.23, 1.11)*
Took urine samples during pregnancy	No (ref)			
	Yes	0.39(−0.01, 0.80)	0.74(0.41, 1.08)*	0.16(−0.16, 0.47)
Took blood samples during pregnancy	No (ref)			
	Yes	1.09(0.56, 1.62)*	0.37(−0.01, 0.76)	0.38 (0.02, 0.74)*
Wealth status	Poorest (ref)			
	Poorer	−0.45(−1.26, 0.37)	0.65 (0.32, 0.99)*	0.48 (0.15, 0.81)*
	Medium	−0.55(−1.35, 0.24)	0.57(0.23, 0.91)*	0.50(0.16, 0.84)*
	Richer	−0.09(−0.72, 0.54)	0.47(0.12, 0.82)*	0.71(0.34, 1.07)*
	Richest	0.05(−0.65, 0.75)	0.71(0.29, 1.13)*	0.61(0.11, 1.11)*
Own television	No (ref)			
	Yes	0.68 (0.36, 1.01)*	0.04(−0.23, 0.32)	0.69(0.35, 1.03)*
Own radio	No (ref)			
	Yes	0.10(−0.17, 0.38)	0.21(0.03, 0.38)*	0.02(−0.18, 0.21)
Electricity	No (ref)			
	Yes	−0.10(−0.59, 0.40)	0.24 (−0.06, 0.54)	−0.15(−0.46, 0.167)
Cons		−4.48(−5.77, −3.18)*	−4.74(−5.58, −3.91)*	−3.96(−4.80, −3.12)*

Note: *P-value < 0.05,* Coeff: Coefficients*,* CI: Confidence interval*

**Table 5:** Elasticity, ECI, and contribution of each determinant for wealth-based inequities of complete CMHS utilization among women who had at least one ANC visit for their recent delivery from 2011 to 2019 in Ethiopia

Variables	Category	2011				2016				2019			
		Elasticity	ECI	Contribution		Elasticity	ECI	Contribution		Elasticity	ECI	Contribution	
				Absolute	Percent (%)			Absolute	Percent (%)			Absolute	Percent (%)
Maternal age in years	< 20 (ref)												
	20–34	0.225	0.099	0.022	10.92	-0.144	0.067	-0.010	-4.91	0.152	0.112	0.017	5.49
	≥ 35	0.057	-0.094	-0.005	-2.62	-0.053	-0.022	0.001	0.59	0.093	-0.070	-0.006	-2.08
	Sum				8.30				-4.52				3.41
Age at first childbirth	< 15 (ref)												
	15–20	-0.087	-0.097	0.008	4.17	0.052	-0.131	-0.007	-3.52	0.028	-0.138	-0.004	-1.25
	≥ 20	-0.058	0.127	-0.007	-3.64	0.085	0.145	0.012	6.38	0.052	0.189	0.010	3.17
	Sum				0.53				2.86				1.92
Sex of HH head	Male (ref)												
	Female	-0.003	0.082	-0.0003	-0.14	0.020	0.041	0.001	0.42	0.007	0.077	0.001	0.17
	Sum				-0.14				0.42				0.17
Residence	Urban (ref)												
	Rural	-0.109	-0.672	0.073	36.04	-0.059	-0.481	0.028	14.62	0.107	-0.621	-0.067	-21.41
	Sum				36.04				14.62				-21.41
Maternal education	No education (ref)												
	Primary	0.024	0.206	0.005	2.46	0.008	0.095	0.001	0.40	0.070	0.069	0.005	1.56
	Secondary/ higher	0.021	0.248	0.005	2.51	0.011	0.294	0.003	1.63	0.066	0.313	0.021	6.62
	Sum				4.97				2.03				8.18
ANC at 1st trimester	No (ref)												
	Yes	0.047	0.178	0.008	4.08	0.093	0.107	0.010	5.12	0.158	0.250	0.040	12.72
	Sum				4.08				5.12				12.72
Parity	1 (ref)												
	2–4	-0.022	0.065	-0.001	-0.71	0.057	0.081	0.005	2.38	-0.141	0.117	-0.016	-5.29
	≥ 5	-0.046	-0.220	0.010	5.00	0.038	-0.187	-0.007	-3.61	-0.061	-0.257	0.016	5.03
	Sum				4.29				-1.23				-0.26
Marital status	Unmarried (ref)												
	Married	-0.110	-0.010	0.001	0.55	-0.160	0.015	-0.002	-1.27	0.079	-0.021	-0.002	-0.52
	Sum				0.55				-1.27				-0.52
Family size	< 5 (ref)												
	≥ 5	0.029	-0.140	-0.004	-1.99	0.035	-0.106	-0.004	-1.88	-0.008	-0.154	0.001	0.40
	Sum				-1.99				-1.88				0.40
Being informed pregnancy complications	No (ref)												
	Yes	0.038	0.205	0.008	3.84	0.121	0.220	0.027	13.69	0.318	0.224	0.071	22.96
	Sum				3.84				13.69				22.96
Caesarean delivery	No (ref)												
	Yes	0.011	0.090	0.001	0.48	0.024	0.066	0.002	0.81	0.035	0.078	0.003	0.89
	Sum				0.48				0.81				0.89
Measured BP during pregnancy	No (ref)												
	Yes	0.215	0.193	0.041	20.41	0.376	0.171	0.064	32.97	-0.072	0.151	-0.011	-3.48
	Sum				20.41				32.97				-3.48
Took urine samples during pregnancy	No (ref)												
	Yes	0.045	0.432	0.019	9.53	0.223	0.249	0.055	28.47	0.251	0.284	0.071	22.85
	Sum				9.53				28.47				22.85
Took blood samples during pregnancy	No (ref)												
	Yes	0.057	0.368	0.021	10.35	0.122	0.219	0.027	13.68	0.311	0.223	0.069	22.34
	Sum				10.35				13.68				22.34
Wealth status	Poorest (ref)												
	Poorer	-0.030	-0.400	0.012	5.82	0.029	-0.370	-0.011	-5.52	0.072	-0.416	-0.030	-9.61
	Medium	-0.036	-0.154	0.006	2.71	0.046	-0.050	-0.002	-1.18	0.067	-0.100	-0.007	-2.15
	Richer	-0.030	0.152	-0.005	-2.24	0.034	0.282	0.010	4.95	0.116	0.216	0.025	8.09
	Richest	-0.042	0.847	-0.035	-17.46	0.074	0.696	0.051	26.46	0.073	0.778	0.057	18.23
	Sum				-11.17				24.71				14.56
Own television	No (ref)												
	Yes	0.026	0.403	0.011	5.23	0.002	0.441	0.001	0.48	0.158	0.613	0.097	31.23
	Sum				5.23				0.48				31.23
Own radio	No (ref)												
	Yes	0.013	0.484	0.006	2.99	0.019	0.379	0.007	3.66	-0.005	0.311	-0.001	-0.46
	Sum				2.99				3.66				-0.46
Electricity	No (ref)												
	Yes	0.032	0.697	0.022	10.84	0.018	0.618	0.011	5.86	0.027	0.770	0.020	6.58
	Sum				10.84				5.86				6.58
Residuals					-9.13				-40.48				-22.08

## Discussion

This study assessed the socioeconomic inequities of complete CMHS utilisation from 2011 to 2019. The overall proportion of each, and combined, maternal healthcare service components was low throughout the surveys. In this study, the recent 2019 EMDHS revealed that among women who had at least one ANC visit during their most recent childbirth, only 58.1% attended ANC4 + visits, and just 29.9% received the complete CMHS. These coverages of maternity care were very far below from the 2030 UHC service coverage target of 80% (World Health Organisation and World Bank, 2021; World Health Organization and World Bank, 2017). This low access and service utilization can be due to women having poor access to healthcare facilities and living in hard-to-reach communities (Jacobs et al., 2012). Unaffordable maternal healthcare costs, including costs of ANC, healthcare facility birthing, and PNC services, might also be a barrier to maternal health service uptake (Banke-Thomas et al., 2020). Although maternity care is officially free at public health facilities in Ethiopia, women incur substantial hidden costs in practice, such as out-of-pocket payments for unavailable medications and diagnostic services. This could be associated with shortages and stockouts of essential supplies at public health facilities (Magunda et al., 2023; Tsegaye et al., 2021). As such, purchasing of medicines and diagnostic services (e.g., laboratory and ultrasound) from private health facilities were unaffordable for poor mothers (Kaba et al., 2015; Wuneh et al., 2022). Fear of high cost of transportation back to their homes were the critical issues for women to attend health facility delivery (Ibrhim et al., 2018; Tiruneh et al., 2021). For example, families of women in labour were reluctant to take critically ill women, whom they believed were likely to die, to health facilities due to the anticipated high transportation costs of bringing deceased bodies back home (Tiruneh et al., 2021). The loss of time during healthcare visits was another key indirect cost affecting maternal healthcare service utilization, as women often prioritized basic needs over seeking care (Gebreyesus et al., 2019; Zeleke & T/Haymanot, 2020).In addition, the low coverage of ANC4 + across the surveys could be due to women’s late initiation of ANC.

Our findings in the recent 2019 EMDHS also indicated that healthcare facility-based childbirth (64.4%) and PNC service utilisation (46.3%) among women who had at least one ANC visit for their most recent birth were notably low compared with the 2030 UHC service coverage target (World Health Organisation and World Bank, 2021; World Health Organization and World Bank, 2017). However, the 2019 EMDHS report shows health facility birthing significantly improved compared with ANC4 + and PNC service utilisation. For example, only 43% and 34% of all pregnant women received ANC4 + and PNC, respectively in 2019, but about 48% of women gave birth in a health facility (CSA, 2019). In comparison, only 10% and 26% of births in 2011 and 2016 surveys had occurred at health facilities, respectively (CSA, 2011, 2016). Moreover, we still observed PNC engagement remained low despite more than 60% of maternal deaths and 47% of child deaths occurring during the early postpartum period (World Health Organization, 2019). Our study also found a high dropout rate between health facility birthing and PNC service utilisation. This finding was consistent with studies conducted in Ethiopia (Geda et al., 2021) and SSA (Bobo et al., 2023). This might be due to poor handling and communication among maternal and child health program personnel to ensure the continuity of care and improved health outcomes for women and children (World Health Organization, 2005).

Complete CMHS showed a pro-rich distribution throughout the surveys. This pro-rich distribution of CMHS implies wealthier women may have better access to, and utilisation of, maternal healthcare services. This also implies a unit change in the proportion of household wealth status of women might have a significant impact on complete CMHS utilisation. This finding was supported by studies conducted in SSA (Asefa et al., 2023; Bobo et al., 2023) and Bangladesh (Methun et al., 2023). This is most likely because wealthier people can afford the costs associated with maternal healthcare services. Conversely, women from households with low wealth status may be unable to pay for healthcare services (Bintabara & Mwampagatwa, 2023). To tackle such challenges, many low-income countries provide fee-exempt maternal healthcare services to curb the disproportionate burden of costs among women from low-household wealth status groups (Hatt et al., 2013). This finding highlights the need for policymakers, program planners, and practitioners in Ethiopia to critically reassess the implementation of the free maternity care policy. For instance, a study conducted in Kenya revealed that free maternity services are often not truly free, as poor women with limited bargaining power are frequently required to make out-of-pocket payments before accessing essential services in public health facilities (Ombere, 2021). In Ethiopia, the fear of unaffordable transportation costs during emergency obstetric care including the need to cover the cost of ambulance fuel and the driver’s per diem may also hinder the utilisation of maternity care services (Alemayehu et al., 2022; Mukuru et al., 2021). In addition, the inability to afford essential delivery items, such as basins, soap, blankets, maternal dressings, and newborn items negatively influence maternal healthcare service utilisation (Uldbjerg et al., 2020). On the other hand, the extent to which facilities lose potential revenue might cause controversial effects regarding the availability of inputs, provider motivation, and quality of services. While fee exemptions can improve access to maternal healthcare services, they can also lead to potential challenges related to provider motivation, quality of care, and financial sustainability (Hatt et al., 2013).

Our study findings also demonstrated wealth-related inequities in utilising complete CMHS across women’s education. Wealth and education levels are interrelated in low-resource settings on influencing maternal healthcare service utilisation (Ahmed et al., 2010; Dimbuene et al., 2018) and our findings also showed similar patterns in coverage of CMHS utilization across women’s education. Education may ensure women’s autonomy, such as freedom of movement, decision-making power, and financial control, which can strongly influence service use and choice (Chowdhury et al., 2007). Our findings were consistent with studies conducted in Ethiopia (Geda et al., 2021), Tanzania (Bintabara & Mwampagatwa, 2023), and SSA (Asefa et al., 2023; Bobo et al., 2023). This might be because initial access to services may be easier for higher-level school-attending women from wealthier groups. As coverage expands and services become more accessible, opportunities for women from poorer groups to access those services also increase (Asefa et al., 2023).

Urban-rural disparities in maternal healthcare service utilisation were observed in every survey period. Complete CMHS and its components were higher among urban women than in rural areas. This result was supported by the findings in Tanzania (Bintabara & Mwampagatwa, 2023), SSA (Bobo et al., 2023), and Bangladesh (Haider et al., 2017; Rahman et al., 2023). This might be because many rural women lack nearby healthcare facilities, requiring long travel times, which can be difficult and costly (Bobo et al., 2023). Inadequate transportation infrastructure (e.g. poor roads) makes travel to healthcare facilities challenging (Centers for Medicare and Medicaid Services, 2019; Syed et al., 2013). Lower levels of education among rural women can also limit their understanding of the importance of maternal healthcare and knowledge of available services (Centers for Medicare and Medicaid Services, 2019). In rural communities, various cultural practices or beliefs may also discourage and restrict women’s autonomy in their decision-making power related to the use of formal healthcare services in favour of traditional remedies or home births (Idris et al., 2023). Similarly, a shortage of health workforces and inadequate supplies in rural health facilities can affect quality care, deterring women from using maternal healthcare services (Centers for Medicare and Medicaid Services, 2019).

This study introduces new approach to measuring wealth-based inequities over time from 2011 to 2019 by examining trends in CMHS across wealth categories. This study gives a valuable evidence for future researchers to explore further the impact of low wealth status on continuity of care. In addition, the study offers methodological insights and encouraging future researchers to assess wealth-based inequities over time for binary health outcome using line graph, CC, and ECI. This study also gives additional methodological insights on how decomposition of concentration index has been conducted to assess service uptake inequities (in this case, complete CMHS) across wealth categories. Importantly, we used ECI to compare the inequities across surveys which is the preferred method of measuring inequities in binary health outcome (Erreygers, 2009). This analysis also have some limitations. In this study, we were unable to specify the time of health check during the PNC visit due to the absence of this data for the 2011 survey. In addition, as is the case with any secondary data analysis, we were constrained by the availability of the variables collected as part of the surveys and the absence of data collection on variables, which may further explain the inequities seen in the population. Missing data and variables were particularly noted for the 2019 EDHS dataset. Another limitation was the absence of direct measures of living standards, such as income or consumption in DHS data, which necessitates the reliance on proxy measures such as household wealth index, which may limit the precision of the analysis. The EDHS data also rely on self-reported data from the survey population, which may introduce recall and social desirability bias.

## Data Availability

This data is from the Ethiopian Public Health Institute division of the Ethiopian Ministry of Health. It is publicly available at: https://www.dhsprogram.com/data/dataset_admin/login_main.cfm.

## Abbreviations

ANC: Ante Natal Care
CMHS: Continuum of Maternal Healthcare Services
CC: concentration Curve
CI: Concentration Index
CSA: Central Statistical Agency
EA: Enumeration Area
ECI: Erreygers Concentration Index
EDHS: Ethiopian Demography and Health Survey
EPHI: Ethiopian Public Health Institute
GCI: Generalized Concentration Index: GLM: Generalized Linear Model: HF: Health Facility
HH: Households
LLMICs: Low and Lower-Middle-Income Countries
MMR: Maternal Mortality Ratio
MoH: Ministry of Health
PNC: Post-Natal Care
RMNCH: Reproductive, Maternal, Neonatal, and Child Health
SDGs: Sustainable Development Goals
SSA: Sub-Saharan Africa
TBAs: Traditional Birth Attendants
WHO: World Health Organization, and UHC: Universal Health Coverage
